# Genome Editing in Mouse Embryo Using the CRISPR/Cas12i3 System

**DOI:** 10.3390/ijms26073036

**Published:** 2025-03-26

**Authors:** Jiale He, Juan Liu, Yuan Yue, Lin Wang, Zhize Liu, Guangyin Xi, Lei An, Jianhui Tian, Yinjuan Wang

**Affiliations:** Frontiers Science Center for Molecular Design Breeding (MOE), Key Laboratory of Animal Genetics, Breeding and Reproduction of the Ministry of Agriculture and Rural Affairs, National Engineering Laboratory for Animal Breeding, College of Animal Science and Technology, China Agricultural University, Beijing 100193, China; jialehe99@163.com (J.H.); liujuanid@163.com (J.L.); yueyuan_1991@163.com (Y.Y.); linwang727@163.com (L.W.); rozikayesuvig@outlook.com (Z.L.); xiguangyin612@163.com (G.X.); anleim@cau.edu.cn (L.A.); tianjh@cau.edu.cn (J.T.)

**Keywords:** CRISPR/Cas12i3, genome editing, *Nanog*, mouse embryo

## Abstract

The CRISPR/Cas system is a sizable family that is currently a popular and efficient gene editing tool. Cas12i3, as a member of the Type V-I family, has the characteristics of recognizing T-rich PAM sequences and being guided by shorter crRNA and has higher gene editing efficiency than Cas9 in rice. However, as a potential tool in accelerating the breeding process, the application of Cas12i3 in mammalian embryos has not yet been reported. Our study systematically evaluated the feasibility of applying CRISPR/Cas12i3 to gene editing in mouse embryos, with the core pluripotency regulator gene *Nanog* as the target. We successfully constructed a *Nanog* loss-of-function mouse embryo model using CRISPR/Cas12i3. At the targeted *Nanog* locus, its editing efficiency exceeded that of the Cas9 system under matched experimental conditions; no off-target phenomenon was detected. Moreover, the Cas12i3 system exhibited no side effect on mouse embryo development and proliferation of blastocyst cells. Finally, we obtained healthy chimeric gene-edited offspring by optimizing the concentration of the Cas12i3 mixture. These results confirm the feasibility and safety of CRISPR/Cas12i3 for gene editing in mammals, which provides a reliable tool for one-step generation of gene-edited animals for applications in biology, medical research, and large livestock breeding.

## 1. Introduction

The clustered regularly interspaced short palindromic repeats (CRISPR)-CRISPR-associated proteins (Cas) system is an immune mechanism of bacteria and archaea against external bacteriophages, which enables prokaryotes to recognize and cleave specific nucleic acids [1]. Utilizing this natural mechanism, the CRISPR-Cas system has been developed as the most popular gene editing tool after zinc finger nuclease (ZFN) and transcription activator-like effector nucleases (TALENs) [2,3,4,5,6]. According to the number and complexity of Cas proteins, the CRISPR-Cas system can be categorized into Class I (Types I, III, and IV) and Class II (Types II, V, and VI) [7]. Class II is currently the most economical and convenient source of gene editing tools due to the homogeneous composition of Cas proteins [8]. Among them, Cas9 is the most representative nuclease system in Types II, containing both conserved HNH and RuvC domains, which together cleave double-stranded DNA [9,10]. The Cas9 system requires the activation of both CRISPR RNA (crRNA) and trans-activating crRNA (tracrRNA), which is complementary to the repeat sequence of the crRNA and promotes the maturation of crRNA [11]. This process allows the Cas9 protein to recognize the PAM (3′ NGG 5′) sequence and its nearby target sequence and usually cleaves double-stranded DNA near 3 bp upstream of the PAM sequence [12,13]. So far, Cas9 has been widely used in animals, plants, and microorganisms, and has become a routine tool for gene editing.

The Type V family, Ih also originated from Class II, has more members, such as Cas12a (Cpf1), Cas12b (C2c1), Cas12c (C2c3), and Cas12g, -h, and –I [14,15,16,17,18]. Among them, Cas12a and Cas12i are different from Cas9; they only need one single RuvC domain to cleave single-stranded DNA, and the single crRNA can complete the maturation process without tracrRNA guidance [16,17,19,20]. The molecular weight of Cas12i is smaller than that of Cas9, Cas12a, and Cas12b, which is beneficial for multi-target plasmids to enter the cell and act simultaneously [16]. It has been shown that wild-type Cas12i proteins are successfully used for in vivo cleavage in mammalian cells [21,22,23]. However, the efficiency is low, in which Cas12i2 is less than 10% for single-site gene editing [24]. In recent years, researchers have continuously optimized the wild-type Cas12i protein, and successively developed xCas12i, Cas12i^MAX^, Cas12^HiFi^, and other nuclease systems [24,25,26]. They successfully performed multi-site gene editing on human, bovine, and porcine cells with gene editing efficiency exceeding 60% and obtained gene-edited pigs by somatic cell nuclear transfer [27]. Recently, Cas12i3, which also belongs to the Type V family, was successfully applied to rice and soybean for multi-site editing with an efficiency of more than 20% [28,29].

However, the application of Cas12i3 in mammal embryos has not been reported. Therefore, our study applied the Cas12i3 system to mouse embryos by microinjection to validate its efficiency and feasibility. We utilized a humanized modified Cas12i3 system to edit mouse zygotes. The results certified that the efficiency of the Cas12i3 system was around 60%; no off-target was detected and longer fragment deletions were generated. At reasonable concentrations, the Cas12i3 system did not affect mouse early embryo development or proliferation of blastocyst cells, ultimately leading to the generation of healthy gene-edited mice. Our work provides data support for more optional tools other than Cas9 and Cas12a to obtain gene-edited animals in a one-step method [30,31].

## 2. Results

### 2.1. Design and Preparation of Mouse Nanog Knockout Using the Cas12i3 System

Nanog is a key gene regulating the pluripotency of early embryos and its functional studies are of significance for the regulation of embryonic stem cells and early embryo development [32,33]. Moreover, it has been demonstrated that the expression of Nanog does not initiate until the morula stage, and its absence does not affect the formation of early blastocysts [34,35]. Therefore, targeting *Nanog* could not only facilitate the investigation of regulatory mechanisms in the first cell fate determination in mice but it could also mitigate the adverse effects of gene modification on early embryonic development and validate the safety of the nuclease system itself.

**Figure 1:**
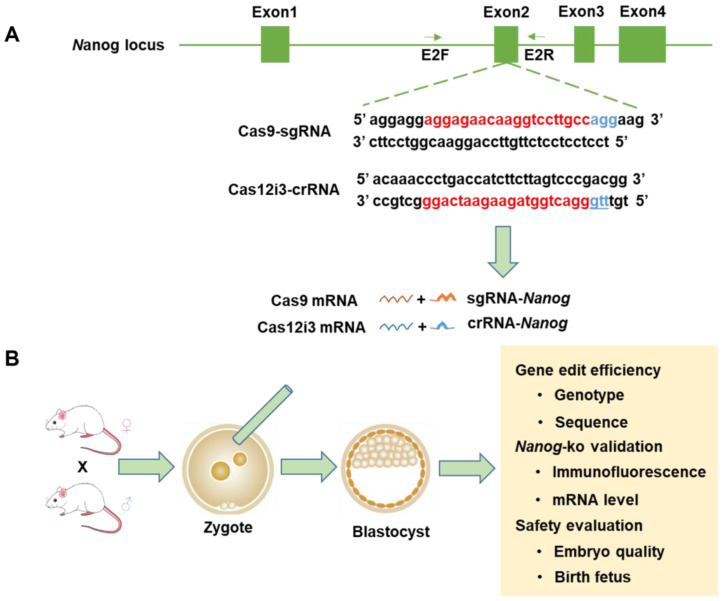
Design and preparation of mouse Nanog knockout using the CRISPR/Cas12i3 and Cas9 systems. (**A**) Schematic diagram of genome editing construction at mouse *Nanog* gene locus by the Cas12i3 and Cas9 systems. The blue sequence is the protospacer adjacent motif (PAM, Cas12i3-TTN, and Cas9-NGG) and the red sequence is the crRNA or sgRNA target recognition sequence. (**B**) Flowchart of the experimental procedure for cytoplasmic microinjection of zygotes and subsequent detection of gene editing effects in mice.

We targeted the second exon of the mouse Nanog and used Benchling, an sgRNA design website recommended by the Zhang Feng Lab, to design and select sgRNAs with the highest score (Figure 1A). We injected zygotes obtained from the oviduct with a nuclease system mixture by cytoplasmic microinjection, and then zygotes were cultured in vitro to the blastocyst stage. Subsequently, we assessed the editing efficacy of different gene editing systems (the Cas12i3 system and the Cas9 system) and evaluated their safety profiles (Figure 1B).

### 2.2. Optimization and Efficacy of the CRISPR/Cas12i3 System in Mouse Embryo

**Figure 2:**
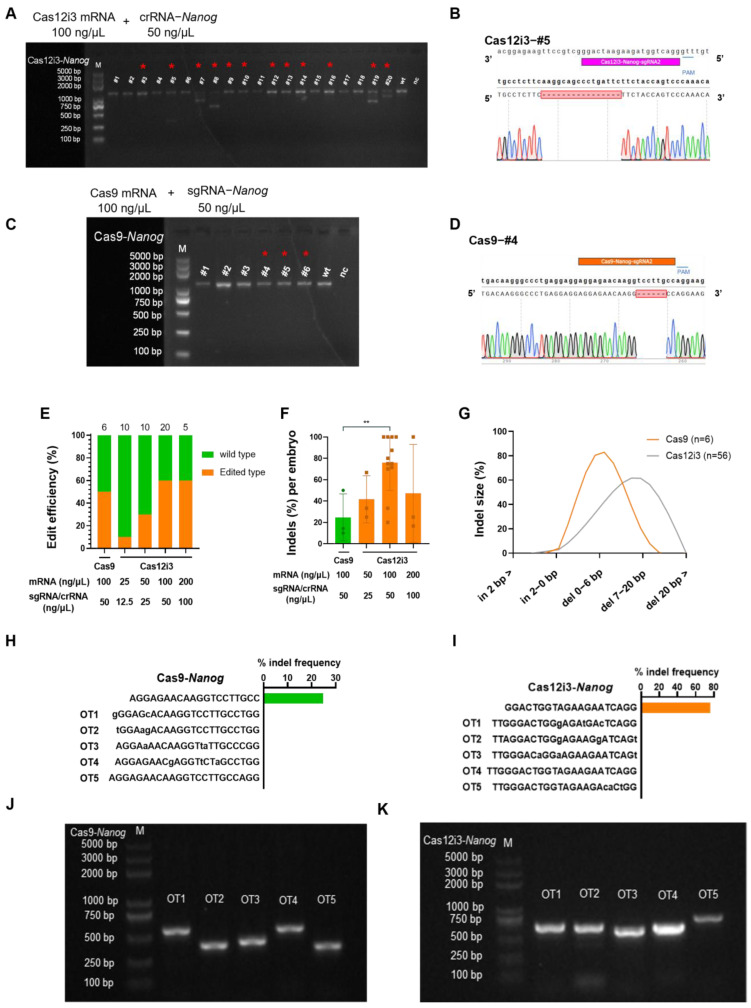
Optimization and efficacy of the CRISPR/Cas12i3 system in mouse embryo. (**A**) PCR amplification of DNA from a single E3.5 blastocyst of the Cas12i3 system. M is a DNA marker. #1 to #20 are the numbers of the 20 E3.5 blastocysts collected. WT is wild type; the results of positive DNA amplification using wild-type E3.5 mouse blastocyst. NC is negative control using water instead of DNA as an amplification template. The precise genome editing outcomes (e.g., insertions, deletions, or substitutions) identified in successfully edited embryos within the #1–#20 cohort are shown in Tables S2 and S3. (**B**) Representative Sanger sequencing chromatograms of Nanog target gene in the Cas12i3 system. Purple markers are expected target sequences and red gaps are missing sequences. (**C**) PCR analysis of DNA fragments near Nanog extracted from a single embryo of the Cas9 system. The precise genome editing outcomes are shown in Tables S3 and S4. (**D**) Representative Sanger sequencing chromatograms of Nanog target gene in the Cas9 group. Orange markers are expected target sequences and red gaps are missing sequences. (**E**) Editing efficiency of the Cas12i3 and Cas9 systems. (**F**) Indel efficiency of a single embryo in the Cas12i3 and Cas9 systems **, *p* < 0.01. (**G**) The statistical trend of indel size generated by the Cas12i3 and Cas9 systems. (**H**,**I**) Potential off-target sites and off-target rates in Cas9 (**H**) or Cas12i3 (**I**) Nanog knockout embryos. (**J**,**K**) Detection of potential off-target sites in Cas9 (**J**) or Cas12i3 (**K**) Nanog knockout embryos.

We first investigated the optimal concentration of the novel nuclease system CRISPR/Cas12i3 in mouse embryos. We set the concentration of Cas12i3 mRNA/crRNA between 25/12.5 ng/μL and 200/100 ng/μL with reference to that of the Cas9 system, which has been widely used. We set up four sets of concentration gradients, and the results showed that the gene editing efficiency was 10% (1/10), 30% (3/10), 60% (12/20), and 60% (3/5) in the 25/12.5 ng/μL, 50/25 ng/μL, 100/50 ng/μL, and 200/100 ng/μL groups, respectively (Figure 2A,B,E and Tables S2 and S3). Here, edit efficiency refers to the proportion of successfully edited embryos relative to the total number of embryos analyzed, including all types of modification. By gradually increasing the concentration of the Cas12i3 system, we observed that within a certain range, the gene editing efficiency improved with a higher concentration of the Cas12i3 mixture. However, when the concentration reached a threshold, like 100/50 ng/μL, further increases in concentration did not enhance the efficiency. When the concentration of the Cas12i3 mixture was excessively high, like 200/100 ng/μL, the injection process became more challenging, and the survival rate of the embryos significantly decreased. Therefore, the highest concentration tested in this study was 200/100 ng/μL of Cas12i3 mRNA/crRNA. Moreover, when the concentration of the nuclease system was too high, it resulted in the generation of multi-genotyped chimeric embryos (Tables S2 and S3), which is inconsistent with the aim of obtaining homozygous gene editing offspring in breeding.

Based on the gene editing efficiency and the type of genotypes generated under different concentrations, the results demonstrated that 100/50 ng/μL of Cas12i3 mRNA/crRNA was the optimal concentration when applied in mouse embryos. Previous works have shown that the Cas9 system targeting a single locus has a high efficiency at 100/50 ng/μL [31,35]. In this study, the gene editing efficiency of 100/50 ng/μL of Cas9 mRNA/sgRNA in mouse embryos was 50% (3/6) (Figure 2C–E and Tables S3 and S4). Subsequently, this concentration was used for both the Cas12i3 system and the Cas9 system if not otherwise specified.

We used the Cas9 system as a control. The indel efficiency of the Cas12i3 system was 76.02 ± 7.52, while that of the Cas9 system was 24.76 ± 12.68 (Figure 2F). Indel efficiency refers to the proportion of insertions or deletions (indels) within a single embryo. This result clarified that the proportion of edited cells in the Cas12i3 group was higher, and the editing effect of Cas12i3 was better at the tested Nanog locus, though system performance may vary across crRNAs or loci. In addition, as shown in Figure 2G, indel size statistics showed that the Cas12i3 system generated 56 indels in 12 gene-edited embryos. Deletions occurring at 0–6 bp accounted for 37.50% (21/56), deletions occurring at 7–20 bp accounted for 60.71% (34/56), and insertions occurring at 0–2 bp accounted for 1.79% (1/56). In contrast, 83.33% (5/6) of the Cas9 group generated fragment deletions occurring at 0–6 bp. Overall, the Cas12i3 system generates relatively longer indel sequences applied in mouse embryos.

Next, we predicted the possible off-target sites of the crRNA or sgRNA we used by using the Cas-OFFinder website. In the predicted sites for Cas9-sg-*Nanog*, one had a mismatch base number of 2, and nine had a mismatch base number of 3. In the predicted sites for Cas12i3-cr-*Nanog*, 16 had a mismatch base number of 3. Five off-target sites with the lowest mismatch number were selected for each sgRNA/crRNA for detection. The results indicated that none of the crRNA or sgRNA had detectable off-targets (Figure 2H–K and Figure S1A,B).

Taken together, these results revealed that 100/50 ng/μL of Cas12i3 mRNA/crRNA exhibited superior gene editing efficiency and generated longer fragment deletions, making it highly suitable in gene knockout experiments.

### 2.3. Construction of the Mouse Preimplantation Embryo Model of Nanog Knockout Using the CRISPR/Cas12i3 System

**Figure 3:**
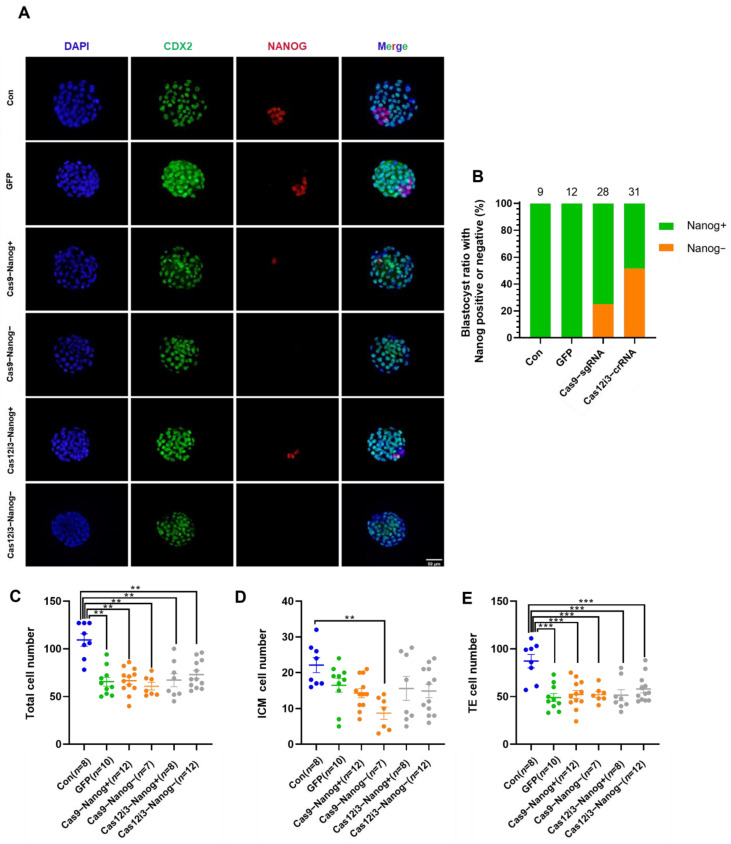
Construction of the mouse preimplantation embryo model of Nanog knockout using the CRISPR/Cas12i3 system. (**A**) Representative immunofluorescence images of E3.5 blastocysts stained for Nanog (red), CDX2 (green), and DAPI (blue). The scale bar is 50 μm. (**B**) Statistics of Nanog immunofluorescence staining results of the Cas12i3 and Cas9 systems in (**A**). Nanog+ indicates that the embryos have positive Nanog signals, and the opposite is defined as Nanog-. (**C**–**E**) Total cell number (**C**), ICM (**D**), and TE (**E**) cell numbers of each group according to staining (**A**). **, *p* < 0.01; ***, *p* < 0.001.

We qualitatively examined the gene editing effects of the Cas12i3 and Cas9 systems at the protein level using immunofluorescence staining (Figure 3A), which showed that some embryos in the Cas12i3 and Cas9 systems were completely devoid of Nanog-positive signals. In contrast, the blank control group and the GFP-injected group expressed Nanog normally, ruling out the potential adverse effects of the microinjection procedure. The statistical results showed that the ratio of Cas12i3-Nanog-(51.61%, 16/31) was higher than that of Cas9-Nanog-(25.00%, 7/28) (Figure 3B). Meanwhile, some embryos contained Nanog-positive cells, although the number of Nanog+ cells was much lower than those in the control group, suggesting these embryos might be chimeras produced by gene editing. The above results demonstrated that the Cas12i3 system effectively edits genes in mouse embryos.

Mouse early embryos undergo their first cell fate decision at the E3.5 early blastocyst stage when they begin differentiating into the trophectoderm (TE) and inner cell mass (ICM) [36,37]. Subsequently, TE develops into the placenta, while ICM gives rise to the fetus [38,39]. The first cell fate decision is crucial for fetal development, and the number of TE and ICM cells is a key indicator for assessing embryo quality and developmental potential. Immunofluorescence staining was performed using CDX2 and Nanog as markers for TE and ICM, respectively. Following the genome editing of Nanog, the number of ICM cells was calculated by subtracting the number of TE cells from the total cell count. The results indicated that the Cas12i3 and Cas9 systems had no effect on the total cell number or the numbers of ICM and TE cells (Figure 3C–E). The observed decrease in cell numbers was attributed to the microinjection procedure. These findings suggest that the first cell fate decision proceeds normally in Nanog knockout mice.

### 2.4. Effects of Nanog Knockdown on Preimplantation Embryo Development in Mice by the CRISPR/Cas12i3 System

**Figure 4:**
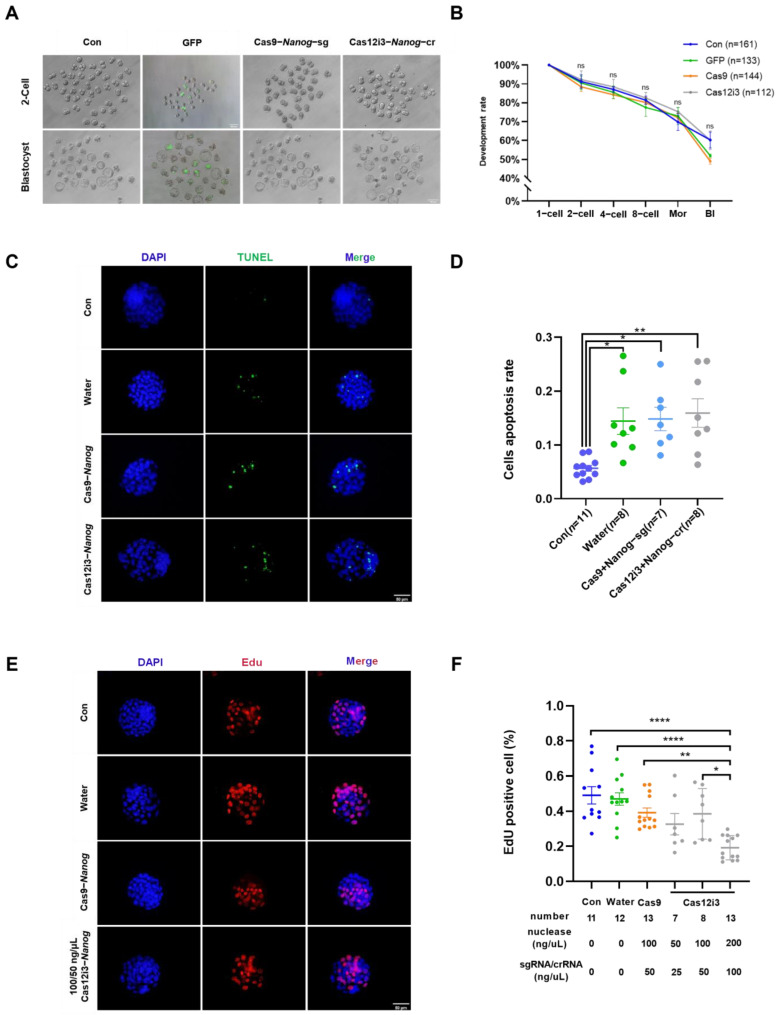
Effects of Nanog knockdown on preimplantation embryo development in mice by the CRISPR/Cas12i3 system. (**A**) Representative images of embryos injected with Cas12i3, Cas9, GFP, or blank control at 2-cell and E3.5 blastocyst stages. The scale is 200 μm and 100 μm, respectively. (**B**) Dynamic statistics of the development rate of early embryos under four different groups. Control group: 5 replicates, *n* = 161 total; GFP group: 4 replicates, *n* = 133 total; Cas9 group: 4 replicates, *n* = 144 total; and Cas12i3 group: 3 replicates, *n* = 112 total. The data represented the mean ± standard error, and ns represented *p* > 0.05. (**C**) TUNEL staining images of E3.5 mouse embryos of different groups with a scale bar of 50 μm. (**D**) The apoptosis rate of embryo cells of different groups based on the staining results of (**C**). (**E**) Representative images of EdU staining of mouse E3.5 embryos of four different groups. The scale bar is 50 μm. (**F**) EdU-positive rate of embryo cells of different groups based on the staining results of (**E**). *, *p* < 0.05; **, *p* < 0.01; ****, *p* < 0.0001.

The development rate, a crucial indicator of in vitro embryo production, is essential for evaluating the impact of external treatments on the developmental potential of embryos. We conducted a preliminary evaluation of the safety of the Cas12i3 system by calculating the embryo development rate at each stage. The dynamic development of early embryos injected with either the Cas12i3 or Cas9 system was recorded at all stages, with data from the GFP-injected group and the untreated group serving as negative and blank controls, respectively. Representative images of each group at the 2-cell and blastocyst stages showed that embryos in all groups displayed similar morphology (Figure 4A). Statistical analysis indicated no significant differences in the development rates of early embryos from the 2-cell to blastocyst stages among the four groups (Figure 4B).

The CRISPR/Cas nuclease system achieves gene editing by causing DSBs to mediate NHEJ junctions in targeted regions. Embryonic stem cells (ESCs) are highly sensitive to DSBs; one single DSB could initiate its own apoptotic program. Mouse ESCs share a lot of similarities with early embryos, and therefore early embryos might have proximate self-correcting mechanisms. We performed a TUNEL assay to assess the level of apoptosis. The results showed that neither the Cas12i3 nor the Cas9 system induced apoptosis in blastocyst cells. However, the microinjection procedure did cause some degree of cell apoptosis (Figure 4C,D). Cell proliferation is highly active during early embryo development, making it a key indicator for assessing both cell and embryo quality. Using an EdU assay, we found that neither the Cas12i3 nor the Cas9 system had a negative impact on the proliferation capacity of blastocyst cells, and the microinjection procedure did not significantly reduce cell proliferation (Figure 4E). We also conducted EdU assays at different concentrations of the Cas12i3 system. The results showed that the proliferation capacity of blastocyst cells was significantly reduced when the concentration increased to 200/100 ng/μL (Figure 4F).

Taken together, these results indicated that the Cas12i3 system is safe for application in mouse embryos and has no negative effect on early embryo development.

### 2.5. Generation of Nanog-Targeted Gene-Edited Mice Using the CRISPR/Cas12i3 System

**Figure 5:**
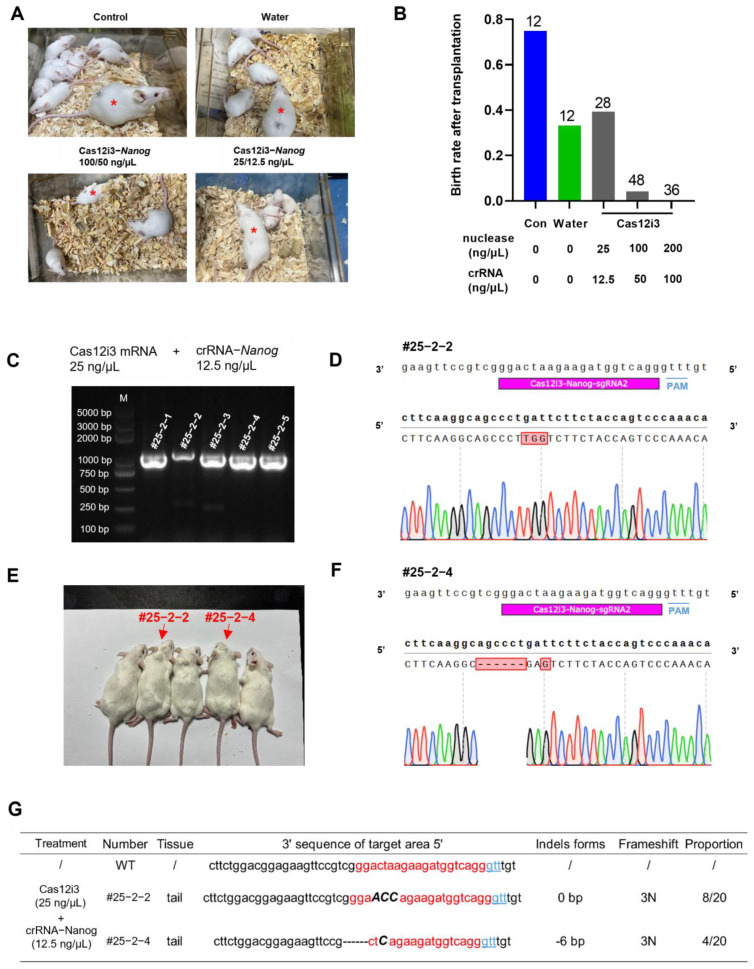
Production of gene-edited mice targeting Nanog using the CRISPR/Cas12i3 system. (**A**) Representative images of gene-edited mice by different concentrations of the Cas12i3 system. Red asterisks represent receptors. Unlabeled mice are transplanted mice. (**B**) The birth rate of the control group and different concentrations of Cas12i3 groups. (**C**) PCR amplification results of mouse tail from gene-edited individuals by the Cas12i3 system. (**D**,**F**) Representative peaks from Sanger sequencing of gene-edited individuals. (**E**) Representative images of gene-edited and wild-type individuals. Red numbers and arrows: gene-edited individuals. (**G**) Specific changes in targeted sites of gene-edited individuals.

A principal aspect of applying new gene editing systems in agriculture, particularly in livestock breeding, is the ability to produce precisely gene-edited individuals. This serves as the most direct measure of the effectiveness and safety of the nuclease system. For this purpose, we injected mouse embryos with varying concentrations of Cas12i3 mRNA/crRNA and observed fetal birth following embryo transfer (Figure 5A). The results showed a significant decrease in the fetal birth rate as the concentration of the Cas12i3 system increased (Figure 5B). At a concentration of 100/50 ng/μL of Cas12i3 mRNA/crRNA, the fetal birth rate was only 4.17% (2/48), whereas reducing the concentration to 25/12.5 ng/μL restored the birth rate to the normal level of 39.28% (11/28) (Figure 5B). Given that Nanog loss-of-function is lethal to embryo implantation, the sharp decrease in birth rate might be attributed to Nanog deficiency. In addition, the microinjection manipulation itself affected embryo quality, leading to a reduction in the birth rate (83.33% vs. 33.33%) (Figure 5B).

To verify the feasibility of the Cas12i3 system in the generation of gene-edited animals, we genotyped individuals born from the 25/12.5 ng/μL of Cas12i3 mRNA/crRNA group. To subsequently explore whether the germ cell line was edited, we extracted genomic DNA from a small amount of tail tissue for PCR, avoiding sampling in multiple organs (Figure 5C). The results showed that the editing efficiency was 18.18% (2/11) under this concentration, which was comparable to the 10% (1/10) detected in embryos. All gene-edited events in the neonates resulted in frameshift mutations in the target gene (Figure 5D,F). Observation of the body condition and activity level of newborn mice indicated that the gene-edited individuals produced by the Cas12i3 system were as healthy as the wild-type mice (Figure 5E). Furthermore, statistical analysis revealed that all gene-edited individuals produced by the 25/12.5 ng/μL Cas12i3 system were homozygous, consistent with the results observed in embryos (Figure 5G).

All these results demonstrated the feasibility and safety of the Cas12i3 system for producing gene-edited mice.

## 3. Discussion

The Cas9 system is widely applied in biomedicine and agriculture due to its high efficiency, low cost, and ease of use [40]. Recently, Type V-Cas12i, which also belongs to Class II, has attracted scientists to participate in the development of novel Cas12i family nuclease systems. This is due to its smaller size, higher gene editing activity, higher fidelity, and broader distribution of PAM (5′ TTN 3′) sequence sites compared to other nuclease systems, like Cas9 and Cas12a [16].

There are many subtypes of the Cas12i protein family; naturally existing Cas12i1 and Cas12i2 nuclease proteins were first discovered and their mechanisms were clearly dissected by protein structure research [17,41]. However, it was found that the efficiency was generally low [17,41]. To solve this problem, researchers modified Cas12i proteins through amino acid replacement, mutation, and so on, to improve its abilities of recognition or cleavage, and successfully screened out xCas12i (hfCas12^Max^), which has high efficiency and high fidelity [25]. Subsequently, with a similar strategy, researchers engineered Cas12i2 using MIDAS technology to develop Cas12i^Max^ with high efficiency and Cas12i^HiFi^ with low off-target rate [24]. Recently, Cas12i^HiFi^ and Cas12i^Max^ have been successfully applied in the cells of livestock animals such as porcine, bovine, and ovine, simultaneously editing multiple genes that control economically important traits, and obtaining gene-edited cloned animals [27]. Furthermore, the efficiency of Cas12i^HiFi^ and Cas12i^Max^ in mammalian cells is significantly higher than that of Cas9, and both have low off-target characteristics [27]. The naturally existing Cas12i3 nuclease protein has been reported to be successfully applied in rice and soybean [23,28,29]. However, it was found that the efficiency of the Cas12i3 system varied greatly for different gene targets in rice [28]. Recently, Duan et al. obtained a Cas12i3 variant, Cas SF01, by modifying the Cas12i3 protein [42]. The verified Cas SF01 was active in rice, pepper, soybean, and mouse liver [42].

Production of CRISPR/Cas9-based gene-edited animals by one-step approach has been well studied over the past decade. Researchers found that the concentration of nuclease mixture in the range of 25–200 ng/μL had no significant effect on the development rate of mouse early embryos [30,31,35,43]. The highest editing efficiency could be 100%, but editing efficiency is only about 40% when injecting a single sgRNA [43]. Therefore, the editing efficiency of the Cas9 system in our study is within the reasonable range and could serve as an effective control treatment.

We explored the gene editing efficiency of different concentrations of Cas12i3 mRNA/crRNA. The editing efficiency was increased with the increasing concentrations of the Cas12i3 system and achieved the peak at 100–50 ng/μL. This trend may be explained by the degradation rate of Cas12i3 protein in cells. According to the Cas9 system, multi-site sgRNA combinations greatly enhance gene editing efficiency, and more attempts could be made to optimize the efficiency of the Cas12i3 system by using crRNA-cocktails [43]. Further studies are warranted to systematically evaluate the Cas12i3 system across multiple loci and crRNA designs, which could comprehensively assess its efficiency relative to Cas9 and other CRISPR systems.

Next, we proved that the Cas12i3 system is active in editing mouse embryos. Given that *Nanog*, the target gene selected for this study, initiates expression during the morula stage and localizes to the inner cell mass at the blastocyst stage [34], this may elucidate the reasons why the percentages of Nanog-negative cells for either the Cas12i3 or Cas9 system are slightly lower than the gene editing efficiency in embryos. The indel efficiency of the Cas12i3 system was 76.02 ± 7.521, which was significantly higher than that of the Cas9 system (24.76 ± 12.68) (Figure 2F). These data suggested that the Cas12i3 system outperforms the Cas9 system at the tested Nanog locus under standardized conditions, though broader validation is required to generalize this observation. In the NHEJ-mediated gene editing experiment, genes are successfully knocked out mainly by disrupting the protein structure through frameshift mutation. Sanger sequencing data shed light on the editing effect of the Cas12i3 system, with a frameshift mutation rate of 78.57% (44/56). Meanwhile, indel size data showed that the Cas12i3 system generated a higher percentage of fragment deletions of over 6 bp than the Cas9 system, indicating that Cas12i3 tended to induce longer indel sequences. These results were consistent with a previous study [27].

To promote the application of the Cas12i3 system in mammals, safety needs to be ensured. At an efficient concentration of 100/50 ng/μL of Cas12i3 mRNA/crRNA, the Cas12i3 system did not affect mouse early embryo development, and the embryos underwent the first cell fate. Gene-edited embryos using the Cas12i3 system shared similarities with the control group in terms of morphology, growth rate, development rate, and the expression of ICM and TE markers. However, due to the specificity of the selected target gene, *Nanog*, its loss-of-function will directly lead to abnormal implantation of mouse embryos [34]. Therefore, the low birth rate of high-concentration groups mainly originated from the aberrant expression of *Nanog* rather than the Cas12i3 system. But we need to note that too high concentrations of Cas12i3 will significantly inhibit the proliferation of blastocyst cells and microinjection manipulation itself will trigger embryo cell apoptosis [34,44]. In the future, the use of less invasive delivery methods for gene editing systems will further improve the survival rate of edited individuals [45]. Surprisingly, the fetal birth rate returned to normal when the concentration of the Cas12i3 system was adjusted to 25/12.5 ng/μL, and healthy, gene-edited mice were obtained. Furthermore, the low concentration of the Cas12i3 system was effective in producing single-genotype individuals, which is of great significance for livestock breeding.

In summary, our study applies the Cas12i3 system in gene editing in early mouse embryos, which strongly demonstrates its effectiveness and safety for gene editing in mammalian embryos and gives specific dosage recommendations for practical applications. The results provide strong support for the development and application of this novel gene editing tool.

## 4. Materials and Methods

### 4.1. Experimental Animals

This study utilized 8-week-old female ICR mice and 10-week-old male ICR mice, procured from SPF (Beijing) Biotechnology Co., Ltd. (Beijing, China). The animals were maintained in optimal conditions with controlled lighting, temperature, and humidity, and had free access to food and water. All experiments were conducted in compliance with the relevant regulations and requirements of China Agricultural University and were approved by its Animal Welfare Committee.

### 4.2. The sgRNA/crRNA Design for Nanog

The sgRNA/crRNA target sequences for the mouse Nanog gene (NC_000072.6) were designed using Benchling (https://www.benchling.com/ (accessed on 5th September 2022)), one of the CRISPR design tools recommended by the Zhang Feng Lab (Cambridge, MA, USA). The on-target highest-score sgRNA (20 bp, 3′ NGG 5′ PAM sequence) and crRNA (20 bp, 5′ TTN 3′ PAM sequence) were selected [15]. Specific sequence details are provided in the Supplementary Materials, Table S1, and synthesis was entrusted to Shanghai Sangon Biotech Co., Ltd. (Shanghai, China).

### 4.3. Construction of sgRNA/crRNA Expression Vectors

The vectors PX330 (addgene) and pPL669-PB-hu6-Cas12i.3 (gift from Professor Sen Wu, China Agricultural University, Beijing, China) were linearized using the restriction enzyme BbsI-HF (NEB, Ipswich, MA, USA, R3539S) and purified. The synthesized sgRNA/crRNA primers were diluted, annealed, and ligated with the linearized vectors using T4 DNA Ligase (Thermo Scientific, Waltham, MA, USA). The ligated products were transformed into FAST-T1 competent cells (Vazyme, Nanjing, China, C505) and sequenced for verification.

### 4.4. In Vitro Transcription of crRNA/sgRNA and Cas12i3 mRNA

T7 promoter sequences were added to the primers (Supplementary Materials, Table S1) for PCR amplification. pPL657-CMV-Cas12i.3 (humanized)-2A-Puro (gift from Professor Sen Wu, China Agricultural University) was used as a PCR template for Cas12i3 mRNA. The in vitro transcription was performed using MEGAshortscript™ Kit (Invitrogen, Carlsbad, CA, USA, AM1354) and mMESSAGE mMACHINE™ T7 ULTRA Transcription Kit (Invitrogen, AM1345), followed by purification using MEGAclear™ Transcription Clean-Up Kit (Invitrogen, AM1908). Cas12i3 mRNA and Cas9 mRNA (previously stored in the laboratory) were mixed with their respective crRNA and sgRNA. The end concentration of nuclease with sgRNA/crRNA was adjusted to 100 ng/μL and 50 ng/μL, respectively, and stored at −80 °C.

### 4.5. Mouse Embryo Collection and In Vitro Culture (IVC)

Eight-week-old female mice in good condition were selected and intraperitoneally injected with 0.1 mL of Pregnant Mare Serum Gonadotropin (PMSG) (5 IU) to induce estrus synchronization. After 44–48 h, the mice were injected with 0.1 mL of Human Chorionic Gonadotropin (hCG) (5 IU) to induce superovulation. The superovulated female mice were mated with male mice. Vaginal plugs were checked the following morning to identify mated females, which were then separated. Approximately 18 h post-hCG, the female mice were euthanized by cervical dislocation, disinfected with 75% ethanol, and the abdominal cavity was opened surgically to collect the oviducts. The oviducts were placed in a pre-warm M2 medium. Using a 1 mL syringe needle, the ampulla of the oviducts was incised to allow the cumulus–oocyte complexes (COCs) to flow out naturally. The COCs were transferred using a mouth pipette to 0.1% hyaluronidase for about 3 min to remove cumulus cells, and the denuded zygotes were then transferred to the M2 medium. Fertilized eggs with extruded polar bodies were selected and washed three times in equilibrated KSOM medium before being cultured in KSOM droplets at a density of 30 embryos per droplet. The embryos were incubated at 37 °C in a 5% CO_2_ incubator.

### 4.6. Embryo Cytoplasmic Microinjection

Control of injection pressure was achieved using the FemtoJet microinjector (Eppendorf, Hamburg, Germany) with specific parameter settings: pressure constant/compensation pressure (PC) was set to 10–17 hPa, injection pressure (Pi) to 150 hPa, and injection time (ti) to 0.3–0.6 s. The injection solutions were categorized into three types: GFP mRNA, Cas9 mRNA/sgRNA, and Cas12i3 mRNA/crRNA. The embryos were then cultured post-injection to assess the developmental impact of different nucleases.

### 4.7. Single Embryo DNA Extraction

Blastocysts from each group were collected and transferred to individual embryos into 200 μL microcentrifuge tubes. The Discover-sc Single Cell WGA Kit V2 (Vazyme, SC101) was used to perform high-fidelity amplification of genomic DNA following the manufacturer’s protocol. The products were diluted to a concentration of 100 ng/μL for subsequent use.

### 4.8. Mouse Tail DNA Extraction

Amounts of 1–3 mm of tail tip tissue from 3-week-old mice were cut and placed into individual 1.5 mL microcentrifuge tubes. DNA was extracted by using the One Step Mouse Genotyping Kit (Vazyme, PD101-01) according to the manufacturer’s protocol. The tubes were incubated in a metal bath at 55 °C for 30 min to allow lysis, followed by inactivation of Proteinase K at 95 °C for 5 min. Finally, the samples were mixed thoroughly by vortexing and centrifuged briefly to collect the contents.

### 4.9. Mouse Embryo Transfer

Fertilized eggs extracted from the oviducts were microinjected and cultured in vitro in the morning, and then healthy female mice were selected (characterized by a red vulva) and mated with vasectomized male mice. The next morning, the female mice were checked for vaginal plugs, and those exhibiting plugs were selected as recipients.

On the day of embryo transfer, morphologically normal E3.5 blastocysts were selected and placed in a pre-warmed M2 medium. The warming plate (37 °C) and anesthetic were preheated in advance. Each recipient mouse was administered an intraperitoneal injection of 0.5 mL of anesthetic. Once the mice ceased movement and showed no eye reflex upon stimulation, they were ready for the embryo transfer procedure. Surgical instruments (forceps and ophthalmic scissors) and the operating table were also sterilized in advance.

The anesthetized mice were placed dorsally on the operating table and disinfected with 75% alcohol. The fur was incised using ophthalmic scissors at the junction of the spine and the last rib. The scissors were then replaced to make a small incision in the muscle. Using forceps, one uterine horn was gently extracted. A small puncture was made near the junction of the uterus and oviduct using a 1 mL syringe. A glass needle connected to a mouth pipette was inserted through the puncture, and no more than six embryos were deposited into one uterine horn. The uterus was then carefully returned to the abdominal cavity by holding the fat pad. The same procedure was performed on the opposite uterine horn. Finally, the incision was sutured completely. The recipient mice were placed on a warming plate until they regained consciousness and then returned to the cage.

### 4.10. Detection of Gene Editing Efficiency and Types

For each group, blastocyst-stage embryos were individually collected into 200 μL tubes. Genomic DNA was amplified using the Discover-sc Single Cell WGA Kit V2 (Vazyme, SC101). Target sequences were PCR-amplified with primers and Tks Gflex™ DNA Polymerase (Takara, Beijing, China, R060). PCR products were analyzed using agarose gel electrophoresis to visualize their size, and then cloned into a blunt-end vector via the Ultra-Universal TOPO Cloning Kit (Vazyme, C603), and then transformed into competent cells. At least 10 colonies per plate were sequenced by Sangon Biotech using M13F/R primers. Sequencing data were analyzed with SnapGene 7.0.2 to identify gene editing types.

### 4.11. Off-Target Effect Prediction and Detection

The online prediction tool Cas-OFFinder (http://www.rgenome.net/cas-offinder/ (accessed on 5th January 2024)) was utilized to predict potential off-target binding sites for Cas9-sgRNA and Cas12i3-crRNA [16]. The top five sites with the fewest mismatched bases were selected, and corresponding primers were designed for these sites (Supplementary Materials, Table S5).

### 4.12. Embryo Immunofluorescence Staining

Embryos at the blastocyst stage were collected and their zona pellucida was removed using 0.1% acidic Tyrode’s solution before being washed in PBS-PVA. They were fixed in 4% paraformaldehyde for over 2 h, followed by washes and permeabilization in 0.5% Triton X-100. After permeabilization, the embryos were blocked in 1% BSA with Triton X-100 and then incubated overnight with Nanog (Cell Signaling Technology, Danvers, MA, USA, 8822T, Rabbit) and CDX2 (BioGenex, Fremont, CA, USA, MU392A-UC, Mouse) primary antibodies at 1:1000 dilutions. After washing, they were incubated with secondary antibodies: Goat anti-Rabbit Alexa Fluor 594 (Invitrogen, A-11034) and Goat anti-Mouse Alexa Fluor 488 (Invitrogen, A-11030), each at 1:1000 dilutions, followed by additional washes. Finally, they were placed on glass slides with DAPI-containing mounting medium, and then stored at 4 °C away from light.

### 4.13. TUNEL Staining

Apoptosis of blastocyst cells was detected using the one-step TUNEL apoptosis detection kit (Beyotime, Shanghai, China, C1088). The blastocysts were prepared as immunofluorescence staining, the zona pellucida was removed, and denuded blastocysts were fixed, permeabilized, and washed. The TUNEL reaction mixture (Beyotime, C1088) was prepared and added to the blastocysts, which were incubated at room temperature for 1 h in the dark, followed by three washes (all subsequent steps were performed under dark conditions). Nuclear staining was performed using DAPI, and coverslips were applied. The proportion of apoptotic cells in individual embryos was quantified by merging DAPI and TUNEL-stained images using ImageJ 1.53k, followed by counting the total number of cells and the number of cells with co-staining signals and calculating their ratio.

### 4.14. EdU Staining

The proliferation of blastocyst cells was assessed using the BeyoClick™ EdU-594 Cell Proliferation Detection Kit (Beyotime, C0078S). The EdU staining solution was prepared by diluting EdU dye at a 1:500 ratio in KSOM medium, forming culture droplets in a 3.5 cm dish, which were equilibrated in an incubator for at least 30 min prior to use. Blastocysts were transferred from normal KSOM culture droplets to the equilibrated EdU-containing medium and incubated for 1–2 h in the incubator. The blastocysts were washed three times in 0.1% PBS-PVA droplets and then fixed at room temperature. Following three washes, the blastocysts were transferred to 0.5%-Triton X-100 and permeabilized on a shaker for 30 min. The Click reaction solution was prepared in the dark, and the samples were washed three times, incubated in the Click reaction solution for 30 min, and then washed three times promptly. All subsequent steps were performed under dark conditions. Next, the blastocysts were transferred to 0.5%-Triton X-100 and permeabilized on a shaker for 30 min and then washed three times. Nuclear staining with DAPI and coverslip mounting were performed. The proportion of EdU-positive cells in individual embryos was quantified by merging DAPI and EdU-stained images using ImageJ. The total number of cells and the number of cells co-stained with both signals were counted, and their ratio was calculated.

### 4.15. Data Statistics and Analysis

Data from this study were processed using Excel and GraphPad Prism 8. The significance between two groups of data was analyzed using the two independent samples T-test, while the significance among multiple groups was assessed using one-way ANOVA, with *p*-value thresholds for significance (*: 0.01 < *p* < 0.05 and **: 0.001 < *p* < 0.01).

## Data Availability

Data are included in the article and the Supplementary Materials.

## Supplementary Materials

The following supporting information can be downloaded at https://www.mdpi.com/article/10.3390/ijms26073036/s1.

## Abbreviations

The following abbreviations are used in this manuscript:

CRISPR	Clustered regularly interspaced short palindromic repeats
ZFN	Zinc finger nuclease
TALENs	Transcription activator-like effector nucleases
ICM	Inner cell mass
TE	Trophectoderm
ESC	Embryonic stem cell
