# Supplementary material for: Genome Editing in Mouse Embryo Using the CRISPR/Cas12i3 System
Source: Int J Mol Sci. 2025 Mar 26;26(7):3036. doi: 10.3390/ijms26073036 (PMC11988942; doi:10.3390/ijms26073036)
Supplement: Supplementary file 1 [file ijms-26-03036-s001.zip › ijms-3525276-supplementary.pdf]

Figure S1. Sanger sequencing chromatograms of off-target sites OT1-OT5 of CRISPR/Cas9 (A) and CRISPR/Cas12i3 (B) systems

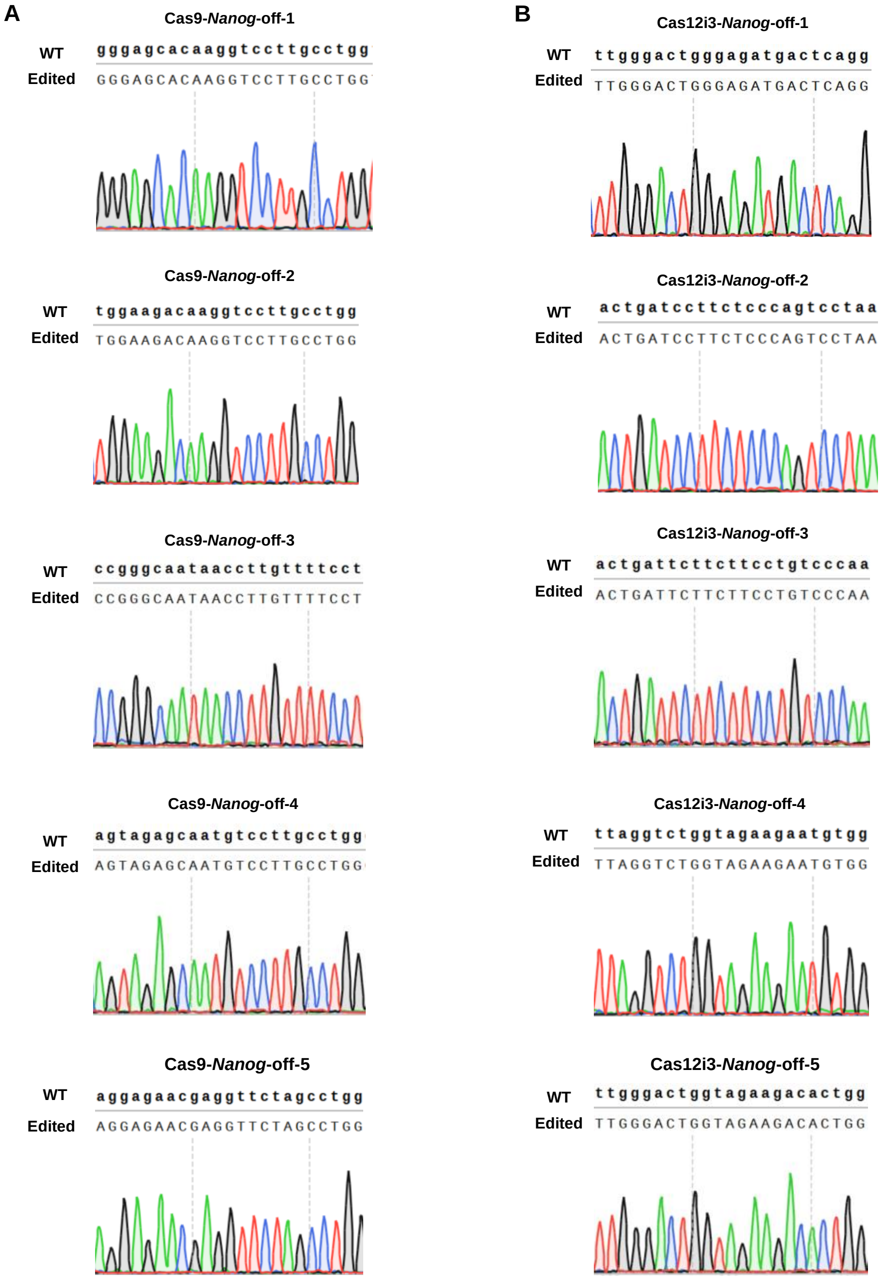

Table S1. sgRNA/crRNA sequences and in vitro transcription template amplification primers

Primers name	5'-3' Sequence
Cas9-*Nanog*-sg-F	caccgAGGAGAACAAGGTCCTTGCC
Cas9-*Nanog*-sg-R	aaacGGCAAGGACCTTGTTCTCCT
Cas12i3-*Nanog*-cr-F	acacGGACTGGTAGAAGAATCAGG
Cas12i3-*Nanog*-cr-R	aaaaCCTGATTCTTCTACCAGTCC
IVT-Cas9-*Nanog*-sg-F	TTAATACGACTCACTATAGGAGAACTATTCTTGCTTACAA
IVT-Cas9-*Nanog*-sg-R	AAAAAAAGCACCGACTCGGTGCC
IVT-Cas12i3-*Nanog*-cr-F	TTAATACGACTCACTATAGGCTCTGACCACCTGAGAGAAT
IVT-Cas12i3-*Nanog*-cr-R	AAAAAAACCTGATTCTTCTACCAGTCC

Table S2. Examples of *Nanog* gene mutations generated by CRISPR/Cas12i3 system

Treatment	Number	3' sequence of target area 5'	Indels forms	Frameshift	Proportion
/	WT	cttctggacggagaagttccgtcgggactaagaagatggtcagggtttgt	/	/	/
Cas12i3 (25 ng/μL) + crRNA-*Nanog* (12.5 ng/μL)	#7	cttctggacggagaagttccgt-------aagaagatggtcagggtttgt	-7bp	3N+1	2/5
Cas12i3 (50 ng/μL) + crRNA-*Nanog* (25 ng/μL)	#3	cttctggacggagaagttccgt--------------gaagatggtcagggtttgt	-9bp	3N	1/3
	#6	cttctggacggagaagttccg------gactaagaagatggtcagggtttgt	-4bp	3N+1	1/4
	#9	cttctggacggagaagttccgtc**A**-----------------gatggtcagggtttgt	-12bp, +1bp	3N+1	2/3
Cas12i3 (100 ng/μL) + crRNA-*Nanog* (50 ng/μL)	#3	cttctggacggagaagttccgtc--------taagaagatggtcagggtttgt	-5bp	3N+2	1/7
		cttctggacggagaagttccgtcggg-------gaagatggtcagggtttgt	-5bp	3N+2	3/7
		cttctggacggagaag-----------------------------atggtcagggtttgt	-19bp	3N+1	1/7
	#5	cttctggacggagaag-----------------------------atggtcagggtttgt	-19bp	3N+1	6/8
		cttctggacggagaag--------------------------ttcatggtcagggtttgt	-16bp	3N+1	1/8
	#7	cttctggacggagaag-----------------------------atggtcagggtttgt	-19bp	3N+1	4/4
	#8	cttctggacggagaag-----------------------------atggtcagggtttgt	-19bp	3N+1	3/4
	#9	cttctggacggagaag-----------------------------atggtcagggtttgt	-19bp	3N+1	4/5
	#10	cttctggacggagaagttccgtc---------aagaagatggtcagggtttgt	-6bp	3N	4/5
		cttctggacggagaagttccgtc--------------aagatggtcagggtttgt	-9bp	3N	1/5
	#12	cttctggacggagaagttccg----------ctaagaagatggtcagggtttgt	-6bp	3N	1/8
		cttctggacggagaagttccgtc---------taagaagatggtcagggtttgt	-5bp	3N+2	1/8
		cttctggacggagaagtt***G***-g--------------agaagatggtcagggtttgt	-10bp, m1	3N+1	3/8
		cttct*A*gacggagaagtt**GG**-------------agaagatggtcagggtttgt	-12bp, +2bp, m1	3N+1	1/8
	#13	cttctggacggagaagttccg--------actaagaagatggtcagggtttgt	-5bp	3N+2	4/7
		cttctggacggagaagttccgt-------------agaagatggtcagggtttgt	-8bp	3N+2	3/7
	#14	cttctggacggagaagttccgtc---------taagaagatggtcagggtttgt	-5bp	3N+2	3/10
		cttctggacggagaagttccgtc----------aagaagatggtcagggtttgt	-6bp	3N	4/10
	#16	cttctggacggagaagttccg--------------agaagatggtcagggtttgt	-9bp	3N	5/5
	#19	cttctggacgga-***G***agt------------------aagaagatggtcagggtttgt	-13bp, m1	3N+1	2/6
	#20	cttctggacggagaagttccgtcgggactaaga*T*gatggtcagggtttgt	m1	/	1/5
Cas12i3 (200 ng/μL) + crRNA-*Nanog* (100 ng/μL)	#1	cttctggacggagaagttcc-*G*c---actaagaagatggtcagggtttgt	-4bp, m1	3N+1	5/5
	#2	cttctggacggagaagttccgtc---------taagaagatggtcagggtttgt	-5bp	3N+2	1/4
	#3	cttctggacggagaagttccgtc----------------------tggtcagggtttgt	-13bp	3N+1	1/6

Table S3. Percentages of different mutation types generated by CRISPR/Cas9 system and CRISPR/Cas12i3 system

Treatment	Mutation type of mouse blastocysts (%)			Indels (%)
	homozygosity	mosaic	WT	
Cas9 (100 ng/μL) + sgRNA-*Nanog* (50ng/μL)	0 (0/6)	50 (3/6)	50 (3/6)	24.76
Cas12i3 (25 ng/μL) + crRNA-*Nanog* (12.5 ng/μL)	0 (0/10)	10 (1/10)	90 (9/10)	4.00
Cas12i3 (50 ng/μL) + crRNA-*Nanog* (25 ng/μL)	0 (0/10)	30 (3/10)	70 (7/10)	12.50
Cas12i3 (100 ng/μL) + crRNA-*Nanog* (50ng/μL)	10 (2/20)	50 (10/20)	40 (8/20)	76.02
Cas12i3 (200 ng/μL) + crRNA-*Nanog* (100 ng/μL)	20 (1/5)	40 (2/5)	40 (2/5)	28.33

Table S4. Examples of Nanog gene mutations generated by CRISPR/Cas9 system

Treatment	Number	5' sequence of target area 3'	Indels forms	Frameshift	Proportion
Cas9 (100 ng/μL) + sgRNA-*Nanog* (50 ng/μL)	WT	tgacaagggccctgaggaggaggagaacaaggtccttgccaggaag	/	/	/
	#4	tgacaagggccctgaggaggaggagaacaagg-------ccaggaag	-6bp	3N	2/8
		tgacaagggccctgaggaggaggagaacaaggtcct-gccaggaag	-1bp	3N+1	2/8
	#5	tgacaagggccctgaggaggaggagaaca-----------------ggaag	-12bp	3N	1/7
	#6	tgacaagggccctgaggaggaggagaacaaggtccttgccCggaag	m1	3N+1	1/10

Table S5. Potential off-target sites and detection primers for CRISPR/Cas12i3 and Cas9 systems

Primers name	5'-3' Sequence	size/bp
Nanog-9-off-1F	TCCTGGATCCAAGCCTAGTG	569
Nanog-9-off-1R	GCTAGCGGCAGACTTATCTGT	
Nanog-9-off-2F	GCCCACAAACCAATTGGACA	400
Nanog-9-off-2R	CCAGGTGGCTTGTGATTAGAG	
Nanog-9-off-3F	TTAAATTGTTTAACCTGGGGCCTG	429
Nanog-9-off-3R	AGGCAAAACCCTTCACCTGTTA	
Nanog-9-off-4F	CAGATTTTGGGAGGTGCTGC	557
Nanog-9-off-4R	GAGCGAATGAGCAGCGGATA	
Nanog-9-off-5F	AGAGAAACAGATAACAGTGGCAAAC	369
Nanog-9-off-5R	GCAGGCCGAGTCTTGTCAAA	
Nanog-i3-off-1F	GAGAACAGTGGCTTTCCTCACA	692
Nanog-i3-off-1R	ATCACAGCAGTTACTCGTCCT	
Nanog-i3-off-2F	AGTGAAAGGTTCCGTACGCC	691
Nanog-i3-off-2R	AGACACGGAGTCTATTGGGC	
Nanog-i3-off-3F	GGGCAAGCTGTTTACCTTCG	631
Nanog-i3-off-3R	AAACCTTTAAAATGCACAGGGCT	
Nanog-i3-off-4F	CCGTCTCAGCTAGTCTTTGTTCT	551
Nanog-i3-off-4R	TTCTTCAAGTCGGGGCCTTTT	
Nanog-i3-off-5F	CAGCTCAGACCTAGGACCCTT	833
Nanog-i3-off-5R	TCTTAGGCCCCATAAGTTGCG	
